# Editorial: Exploring the impact of music interventions on brain function, behavior, and health, volume II

**DOI:** 10.3389/fnhum.2026.1900598

**Published:** 2026-07-13

**Authors:** Ying Liu, Jiancheng Hou, Jie Chen, Guangyuan Liu

**Affiliations:** 1 School of Music, Southwest University, Chongqing, China; 2 Fujian Normal University, Fuzhou, China; 3 University of Wisconsin-Madison, Madison, WI, United States; 4 Hunan Normal University, Changsha, China; 5 College of Electronic and Information Engineering, Southwest University, Chongqing, China

**Keywords:** behavior, brain function, editorial, health, music intervention

Within the interdisciplinary fields of cognitive neuroscience, health psychology, and clinical medicine, the study of music interventions is a significant frontier for understanding their effects on brain function, behavioral change, and overall health. Of particular interest are the regulatory effects of music on brain activity—both at rest and during functional tasks—and its active role in modulating human behaviors such as emotion, cognition, language acquisition, and social interaction. *This Research Topic* aimed to elucidate the complex interplay between brain activity, behavioral change, and health enhancement resulting from various music-based interventions.

Article 1, “*Modulating satiety with neuro-music: a frequency-specific acoustic approach to appetite control*” (Chang et al.), found that frequency-targeted neuro-music, which entrains beta/gamma cortical rhythms (14–30 Hz and >30 Hz), can selectively prolong subjective satiety and reduce the “urge-to-eat” for approximately 30 min in a pre-dining, metabolically needy state.

Article 2, “*Social engagement, pleasure, and memory in musical reminiscence workshops for individuals with Alzheimer's disease*” (Genguelou et al.), found that nine sessions of musical reminiscence workshops significantly enhanced episodic autobiographical memory, verbal interaction frequency, and daily social engagement in individuals with moderate-to-severe Alzheimer's disease. Meanwhile, the pleasure experienced during the workshops was positively correlated with the frequency of social interactions.

Article 3, “*New music combination promotes neuroimmune homeostasis and stress relief* ” (Liu et al.), adopted a novel four-piece music combination, which restores autonomic balance by increasing heart rate variability (HRV) and decreasing sympathetic tone. It also reduces risk-taking behavior, improves attention, and promotes neuroimmune homeostasis—including decreased cortisol and increased salivary immunoglobulin A (IgA).

Article 4, “*Music therapy in health care practice: promise, pitfalls, and policy implications*” (Lan et al.), reported that while music therapy has strong evidence across dementia, Parkinson's disease, stroke, brain injury, autism, depression, insomnia, and palliative care, its widespread implementation is hindered by methodological heterogeneity, workforce shortages, limited reimbursement, and ethical challenges (including informed consent, autonomy, and cultural sensitivity).

Based on a systematic review of 14 original studies involving 1,363 subjects, Article 5, “*Effects of music on post-stroke sleep disorders and treatment perspectives: review and narrative synthesis*” (Yang et al.), found that music intervention consistently and effectively improved sleep status in patients with post-stroke sleep disorders.

When viewed together under the theme “*Exploring the impact of music interventions on brain function, behavior, and health, volume II*,” these five articles and the latest research on music application based on AI (Wei and He, 2026) share several common themes that amplify their individual contributions.

## 1 Music as a regulator of physiological homeostasis

All five studies, whether directly measuring biomarkers or reviewing clinical outcomes, converge on the idea that music restores balance—neural, autonomic, endocrine, or immune. In the field of evidence-based medicine, the positive effects of music on clinical conditions such as cancer, chronic pain, and endocrine disorders have also been confirmed (Fu et al., 2019; Ginsberg et al., 2022). The collective message is that music's power lies in re-establishing physiological equilibrium after specific doses of music intervention.

## 2 Objective, quantifiable neural, and physiological markers

Unlike earlier qualitative music research, these studies prioritize measurable outcomes such as electroencephalogram (EEG) power spectra, heart rate variability indices, cortisol and IgA concentrations, sleep metrics, and observed emotion/engagement ratings. This shift toward biomarker-based assessment strengthens music therapy's credibility in evidence-based medicine (Eze Pue et al., 2023; Lima et al., 2025).

## 3 State-dependent and context-sensitive effects

A nuanced common finding is that music interventions are not universally effective but instead depend on the physiological or metabolic state of the individual. Article 1's neuro-music worked only in the pre-dining (hungry) state, not after snacking. Article 2's reminiscence workshops required repeated sessions to build memory and social effects. Article 3's stress relief was measured under controlled cognitive tasks. Article 5 notes that post-stroke sleep disorders respond best when music is timed to circadian rhythms. This interdependence underscores the need for personalized, condition-specific protocols—moving beyond one-size-fits-all playlists.

## 4 Bridging basic neuroscience with real-world application

Article 1 translates EEG-identified neural signatures of satiety into an acoustic intervention tested on 600 participants remotely. Article 3 moves from heart rate variability (HRV) mechanisms to a concrete music combination. Article 2 converts episodic memory theory into a structured workshop. Articles 4 and 5 synthesize mechanism-driven evidence into clinical guidelines and policy. Together, they model a translational pipeline from bench (neural oscillations and biomarkers) to bedside (dementia care and stroke rehabilitation) to population health (appetite control and stress relief). The volume explicitly targets major public health challenges: obesity (Article 1), Alzheimer's disease (Article 2), chronic stress (Article 3), healthcare system sustainability (Article 4), and post-stroke disability (Article 5). Each article offers a low-risk, high-accessibility intervention—critical for aging populations and resource-limited settings. Article 4's policy analysis directly addresses why such interventions remain underutilized despite strong evidence from Articles 1, 2, 3, and 5.

Collectively, the five articles published in this volume represent a significant leap forward in understanding how music-based interventions can non-invasively modulate human brain function, behavior, and health. They argue that music is not a single therapy but a family of mechanism-driven interventions—each tailored to a specific neural, behavioral, or health outcome. This volume, “*Exploring the impact of music interventions on brain function, behavior, and health, volume II*,” thus serves as a landmark Research Topic that moves the field from “does music work?” to “how, for whom, under what conditions, and at what cost?”—a question that will define the next decade of research and clinical implementation.

However, the articles in this issue still have the following limitations, including small sample sizes, high heterogeneity of intervention protocols, limited longitudinal evidence, and challenges in terms of reproducibility. In the future, we expect more systematic research to specifically address these limitations. To truly serve the public, music research should not only integrate basic disciplines such as music psychology, cognitive neuroscience, and evidence-based medicine to conduct practical applications of music interventions for different groups but should also better combine artificial intelligence technologies to enhance the model analysis and practical application of music-based interventions.
